# Comment on Peker, T.; Boyraz, B. The Relationship between Resistant Hypertension and Advanced Glycation End-Product Levels Measured Using the Skin Autofluorescence Method: A Case–Control Study. *J. Clin. Med.* 2023, *12*, 6606

**DOI:** 10.3390/jcm12247562

**Published:** 2023-12-08

**Authors:** Fadi Alkhami, Ninon Foussard, Alice Larroumet, Marie-Amélie Barbet-Massin, Laurence Blanco, Kamel Mohammedi, Vincent Rigalleau

**Affiliations:** Endocrinology-Diabetology-Nutrition, Bordeaux CHU and University, 33000 Bordeaux, France; fadikhiyami9@gmail.com (F.A.); ninonfoussard@yahoo.fr (N.F.); alice.larroumet@chu-bordeaux.fr (A.L.); marie-amelie.barbet-massin@chu-bordeaux.fr (M.-A.B.-M.); laurence.blanco@chu-bordeaux.fr (L.B.); kamel.mohammedi@chu-bordeaux.fr (K.M.)

We read with interest the recent article by Peker et al. [1], who measured high skin autofluorescence (SAF) in 88 patients with resistant arterial hypertension, arguing for a role for advanced glycation end-products (AGEs) in this severe condition.

We reported that SAF was a marker of glycemic memory in Type 2 Diabetes, related to previous HbA1c levels and vascular complications in 905 patients [2]. Peker’s report prompted us to test whether or not SAF was also related to arterial hypertension in our patients. Most of them (594/905) had arterial hypertension, and their SAFs were higher: 2.7 ± 0.6 AU vs. 2.6 ± 0.6 for others, *p* = 0.016. This +0.1AU difference was less than the +0.7 difference found by Peker. However, our patients did not all have resistant hypertension, and we did not register those who did not. Hence, the comparison is not apple-to-apple.

Peker and Boyraz interestingly discussed how AGEs could promote arterial hypertension, particularly decreased nitric oxide availability, and higher sodium reabsorption at the proximal renal tubules, which may also occur in patients with diabetes, with a potential interest for treatments using sodium-glucose transport protein 2 inhibitors [1].

We suggest that the high SAF in resistant hypertension may, on the other hand, have been the consequence of some characteristics of their patients. Most of them (47/88) had diabetes, and chronic hyperglycemia is known to promote the production of AGEs [3]. However, the SAFs were still higher after excluding them. As depicted in the Table 4 of the article, the 41 non-diabetic remaining participants were 5 years older than the controls (*p* = 0.003). Because the accumulation of AGEs detected via SAF is a long-term process, this 5-year difference surely contributed to the higher SAF in subjects with resistant hypertension. Koetsier et al. reported that the SAF was related to age in the general population without diabetes [4], and proposed reference values: SAF = (0.023*age) + 0.83. We could therefore expect five-year older subjects to have +0.1AU values, which cannot explain a +0.6 difference. The accumulation of AGEs also depends on their renal clearance, and high values are reported in subjects with chronic kidney disease [5]. Glomerular filtration rates below 60 were an exclusion criterion in Peker’s study. However, due to their higher ages and slightly higher creatinine levels (Table 2), it seems possible that the estimated glomerular filtration rate (eGFR) differed in subjects with resistant hypertension. Although it did not reach significance, their more frequent smoking habit (Table 4) may have contributed to the high SAF; tobacco is known to contain AGEs [6].

Among our patients, arterial hypertension did not significantly relate to the SAF, adjusted for their age, sex, eGFR, and smoking habit. Although this may introduce a collider bias, the conclusions of Peker and Boyraz would be stronger if they could provide a linear regression analysis for the relationship between resistant hypertension and SAF, adjusted for age, sex, eGFR, and smoking habits.
